# Retraction of “Extracellular Vesicles Embedded with Persistent Luminescence Nanoparticles Exhibiting Near-Infrared Emission for Long-Term Tracking in Primary Tumors”

**DOI:** 10.1021/acsaom.4c00295

**Published:** 2024-07-24

**Authors:** Tsai-Lan Liao, Kai-Hung Lin, Fei-Ting Hsu, Ming-Hsien Chan

The authors retract this article (DOI: 10.1021/acsaom.3c00474) because it reports results and uses data from another research team, which were included without their permission. There are also concerns about the reliability and/or misrepresentation of the data presented in Scheme 1, Figure 4, and Figure 5. The article is being retracted due to both concerns.

The original article was published on April 9, 2024, and was retracted on July 24, 2024.

